# Oral health status of Egyptian mothers and their preschool children: association of mother’s oral health literacy and marital satisfaction- a cross-sectional study

**DOI:** 10.1186/s12903-025-06099-8

**Published:** 2025-05-22

**Authors:** Naglaa El-Wakeel, Naglaa Ezzeldein, Amel A. Ramadan

**Affiliations:** 1 Oral medicine and Periodontology Department, Faculty of Dentistry (Girls Branch), Al-Azhar University, Cairo, Egypt; 2 Faculty of Dentistry, October University for Modern Sciences and Arts, Cairo, Egypt

**Keywords:** Marital satisfaction, Oral health literacy, Mothers, Preschool children, Oral health, Caries, Plaque

## Abstract

**Background:**

Oral health literacy (OHL) and marital satisfaction (MS) are known to affect the overall health and quality of life of parents and their children. A possible relation between MS and oral health has never been studied before. We aimed to; first; assess levels of OHL and MS of Egyptian mothers, and second; investigate a possible relation between these levels and the oral health status of the mothers and their preschool children.

**Methods:**

A cross-sectional study was conducted on 130 preschool-aged children and their mothers utilizing a structured, questionnaire consisting of three sections; 1- demographic and oral health-related data; 2- seven questions of ENRICH Marital Satisfaction (EMS) Scale of mothers, and 3-A modified Arabic Rapid Estimate of Adult Literacy in Dentistry (A-REALD-30) to measure the mother’s ability to read 30 commonly used dental terms. Clinical examination of the participants was conducted afterwards to evaluate the oral health status using the Decay, missed filled teeth (DMFT), dmft, plaque, and gingival indices (PI and GI).

**Results:**

The mean (SD) of DMF, PI, and GI for mothers were 5.07(3.41), 1.29(0.45), and 1.13(0.52) respectively. Children’s mean (SD) dmft was 5.42(3.27), the mean (SD) of total EMS and A-REALD-30 scores were 23.41(6.79) and 21.65(6.02) respectively. There was a positive correlation between the EMS score and A-REALD-30, *p*-value=0.44, and *r*=0.068, a negative correlation between EMS score and DMFT, dmft, PI, and GI, *p*-value=0.0043, 0.032, 0.956, and 0.730, and *r*=0.253, 0.188, 0.005, and 0.031, respectively. Whereas A-REALD-30 showed a positive correlation with the DMFT and dmft, and a negative correlation with PI and GI *p*-value=0.549, 0.412, 0.158, and 0.670, and *r*=0.053, 0.073, 0.125, and 0.038, respectively.

**Conclusions:**

Within our limitations, this work showed for the first time that there is a possible association between MS and oral health status of mothers and their preschool children. Further, this work unexpectedly showed a positive relation between A REALD-30 and DMFTs implying that REALD-30 alone would not be enough and is suggested to be used with other tools to assess OHL levels. Further long-term studies are needed to clarify how literacy and marital status affect oral health.

**Supplementary Information:**

The online version contains supplementary material available at 10.1186/s12903-025-06099-8.

## Background

Oral health literacy (OHL), is a main determinant of oral health; defined as the individual’s ability to access, process, and understand basic oral health information; access basic oral health services, and make appropriate oral health decisions [1]. Limited OHL is linked to an increased prevalence of oral diseases, poor oral health behaviors, unsatisfactory oral health outcomes, and reduced utilization of oral health services. OHL is one of the reasons; why preventable diseases like periodontal diseases and caries remain so common; and why people do not adopt practices like teeth brushing and flossing that were proven effective in maintaining oral health [2, 3]. Several methods are used to measure OHL, including word recognition (e.g., REALD-99 and REALD-30) or comprehension and functional skills-based tools (e.g., TOFHLID). These methods were utilized in a variety of settings and among different populations worldwide, and REALD-30 is the most widely used tool [4].

Another key factor of the family members’ overall health and quality of life is Marital satisfaction [5, 6]. Being linked to couples’ physical and emotional health, having a satisfying marriage results in better management of life’s pressures, better adjustment, and fewer health problems for the parents and their children [7, 8]. Marital satisfaction encompasses aspects arising from reciprocal giving between spouses such as trust, affection, and companionship. The ENRICH Marital Satisfaction (EMS) Scale is widely used as a reliable tool to assess marital satisfaction [9].

Caries and periodontal diseases are the most common oral diseases, both are caused by dental plaque accumulation. According to the WHO Global Burden of Diseases (2023), An estimated 2 billion people suffer from permanent teeth caries, and 514 million children suffer from primary teeth caries. Egypt is a densely populated (over 100 million people), low-middle income country, in which the out-of-pocket payments account for over 60% of total health expenditures [10]. 74% of Egyptian children had dental caries with a mean dmft of 3.23, which was inversely correlated with socioeconomic status and parental education [11]. Further**,** only 7% of Egyptians have healthy gums and gingival bleeding on probing and calculus formation were more common among females [12]. In the developing countries, socio-economic factors are crucial in negatively impacting oral health. Furthermore, healthcare-related challenges faced by Egyptians such as lack of access to healthcare facilities necessitate studying different factors that might affect access to effective prevention programs [12].

OHL is an emerging topic in research, and previous studies showed a relationship between Patients’ OHL levels and caries or periodontal diseases [13, 14]. Moreover, Evidence shows the importance of the family environment in establishing good oral and general health in children, as basically, parents are responsible for children’s various aspects of life. In early childhood, parental supervision in the evolution of tooth brushing skills and the control of sugar intake, were the most effective preventive measures for dental caries and plaque accumulation [15].

Generally, limited data are available on the effects of OHL on the oral health status of parents and their children, and these data are even more limited on the OHL among the Egyptian population [3, 16–18]. Although marital satisfaction is one of the main determinants of health state valuation in Egypt and worldwide [19, 20], the effect of marital satisfaction on oral health status was not considered for investigation before. Our hypothesis was that; as marital quality affects many of both parent’s and children’s health aspects, it would also affect their oral health status. This study aims to evaluate the impact of maternal oral health literacy and marital satisfaction on the oral health status of both mothers and their preschool children.

## Methods

### Study design and study settings

This cross-sectional study was conducted from December 2023 to March 2024. Participants were selected from the pediatric dentistry clinics of Faculties of Dentistry, Azhar University (Girls branch), and October University for Modern Sciences and Arts. The research proposal was approved by the Research Ethics Committee of the Faculty of Dentistry at Al-Azhar University, Girls branch, (protocol code: P-PD-23–30) and following the Helsinki Declaration. The aim and procedures of the study were explained to the mothers and 2 informed consents were obtained from each mother (one for the mother and one for the child). The STROBE guidelines were utilized to guarantee the reporting of this cross-sectional research [21].

### Sample size calculations

To the correlation between the degree of marital satisfaction and A-REALD-30, the Sperman’s correlation coefficient (r) test was applied. As no previous data was available on the tested outcomes, we did a pilot study on 40 subjects using the final approved form of the questionnaires and an effect size ρ=0.253 was detected, so, a minimum total sample size of 120 participants was sufficient to detect the correlation between the degree of marital satisfaction and A-REALD-30 at power (1-β=0.80) and significance probability level of *p* ≤ 0.05. The sample size was calculated according to G*Power software version 3.1.9.7.

### Study participants

The study was conducted on 130 children aged between two and six years and their mothers with the following criteria**.**

### Inclusion criteria

- Children between 2–6 years old and accompanied by their mother.
- Children have been residing with their mother since birth.
- Currently married mother, with no cognitive, vision, or hearing difficulties and who attained secondary or higher education to be sure they can read the REALD and respond to the EMS questionnaires.
- Mothers not working or training in a health care facility.

### Exclusion criteria

- Child who refuses to cooperate during clinical examinations.
- Children with special health care needs.
- Mothers who declined to take part in the research, or did not finish answering the questionnaire.

### Outcomes

This study assessed the impact of maternal oral health literacy and marital satisfaction on the oral health status of both mothers and their preschool children.

### Data collection (study instrument)

Data was collected via a questionnaire and clinical examinations. Both were carried out in a private area and lasted fifteen minutes. The interviews were conducted by two of the investigators (N E and N E) who are experienced and trained interviewers. Before the interview, participants were assured of their anonymity as their responses weren’t linked to any identifying information like name or address

### Questionnaire

A questionnaire was designed to cover the study outcomes guided by previously validated and published studies [22–25]. An independent committee of experts in periodontics, oral public health, and community medicine validated the questionnaire for face, clarity, and content validity and approved the final form. Afterward, a pilot test was conducted on forty participants to ensure that the questions were clear and appropriate. Some questions and answers were modified. In light of previous study [25],questions were removed from the 15-item validated form of the EMS scale, as mothers complained about the length and boredom of the questionnaire that caused them to lose focus in answering. Thus, only 7 questions that cover the main issues in a marriage were selected. The pilot study results were not included in the final analysis. Sample selection bias was avoided by using convenience consecutive sampling.

The questionnaire consisted of three sections:

- Section 1: Included eleven close-ended questions that explored the background characteristics of the mother and her child, including; age of the mother and child, gender of the child, place of residence, mother’s occupation and level of education, source of oral health information, frequency of tooth brushing by mothers and child, number of children’s daily sugar intake, and frequency of dental visits of the mother.
- Section 2**:** Evaluated the mother’s marital satisfaction using the EMS scale [23] Seven closed-ended questions (questions 2, 3, 6, 7, 8, 9, and 12) were chosen from the original scale to measure the degree of satisfaction with the partner’s personality, responsibilities, relationship success, decision-making, financial status, and relationship needs. The scale contains 7 questions scored on a Likert scale ranging from 1 to 5 (strongly disagree to strongly agree) (suppl.1). The total score of this questionnaire varies from 7 to 35. Higher scores indicate higher marital satisfaction.
- Section 3: Evaluated mother’s OHL using a validated Arabic version of the Rapid Estimate of Adult Literacy in Dentistry (A-REALD-30), a verified word recognition test [24]. Each mother was given a list of 30 dental terms (e.g., hypoplasia, plaque, etc.) and had to read the term aloud. Each correct pronunciation scored a point, while hesitation, pauses or repetition received no points. The total score for AREALD-30 ranged from 0 to 30 with a higher score indicating a higher level of OHL.

### Clinical examination

After finishing the questionnaire, clinical examination of both the mother and the child was done. A pedodontist examined all children and periodontist examined all mothers in each faculty with high inter-examiner reliability, using a dental mirror, explorer, and under the dental unit light. Mothers were assessed for decayed, missing, and filled teeth in the permanent dentition (DMFT index), gingival index (GI) and plaque index (PI), using a UNC 15 prob [26, 27]. The children were assessed for the dmft index for the primary dentition [28].

### Statistical analysis

The demographic and oral health-related data of participants, clinical examination, MS score, and REALED-30 data were reported using descriptive statistics (mean, standard deviation, percentage, and number). Sperman’s Correlation test was applied to measure the strength and direction of a linear relationship and the correlation between the tested parameters. Sperman’s correlation is a statistical test used to measure the strength and direction of a linear relationship between two variables. Statistical evaluation was performed using the SPSS statistical package (version 25, IBM Co. USA).

## Results

### Demographic and oral health-related data of participants

In this study, 56.2% of children were females, with a mean age (SD) of 4.86±0.85 years. Nearly half of the mothers (50.8%) were below 30 years old; the rest were above 30 yrs. 65.4% of mothers were housewives, the rest were working mothers. For levels of education, 53.8% held a university degree and the rest attained a secondary school education. 62.3% of participants resided in urban areas. Regarding the source of oral health information, 41.5% of mothers reported from the Internet, 18.5 % from media, and 11% from healthcare-related personals. 30% of children brush their teeth once daily, and 30% of mothers brush their teeth twice daily. 39.2% of the children consume sweets 3 times a day. Most dental clinic visits were not regular follow-ups but emergency visits (93.1%) (Table 1).

**Table 1:** Frequency, percentage of the demographic and oral health-related data of participants

**Personal Data**	***N* (130)**	**%**
Mother’s Age		
< 30	66	50.8
> 30	64	49.2
Child’s sex		
Male	57	43.8
Female	73	56.2
Area of residence		
Urban	81	62.3
Rural	49	37.7
Mother’s occupation		
Working	45	34.6
Not working	85	65.4
Mother’s education level		
Secondary school	60	46.2
University degree	70	53.8
Oral health information source		
Media	24	18.5
Books	3	2.3
Internet	54	41.5
Health services	15	11.5
Others	19	14.6
Nothing	15	11.5
Brushing of the child’s teeth		
Up to 1	39	30.0
≥2	21	16.2
Irregular	36	27.7
No brushing	34	26.2
Brushing of the mother’s teeth		
Up to 1	36	27.7
≥2	39	30.0
Irregular	33	25.4
No brushing	22	16.9
Child’s sugar consumption		
Irregular	45	34.6
≤3 times/day	51	39.2
>3 times/day	34	26.2
Mother’s present dental visits		
Regular	3	2.3
Only with complaints	121	93.1
Didn’t see the dentist	6	4.6

### Clinical examination data of participants

The mean caries index (SD) (DMFT) for mothers was 5.07±3.41, ranging from 0 to 16, while the mean (SD) for the child caries index (dmft) was 5.43±3.27. The mean (SD) PI for mothers was 1.29±0.45, ranging from 0.5 to 2.3, while the mean (SD) GI was 1.13±0.52, ranging from 0 to 2 (Table 2).

**Table 2:** Mean, standard deviation, and range of the clinical examination for mothers and children

**Clinical examination**	**Mean ±SD**	**Range**
Mother Caries Index (D)	2.1±1.63	0 - 6
Mother Caries Index (M)	1.03±1.47	0 - 7
Mother Caries Index (F)	1.94±2.01	0 - 8
Mother Caries Index (DMF)	5.07±3.41	0 - 16
Child caries Index (d)	4.53±3.04	0 - 16
Child caries Index (m)	0.21±0.93	0 - 6
Child caries Index (f)	0.68±1.14	0 - 4
Child Caries Index (dmf)	5.42±3.27	0 - 16
Mother Plaque Index (PI)	1.29±0.45	0.5–2.3
Mother Gingival Inex (GI)	1.13±0.52	0–2

### Mother’s marital satisfaction

The Mean ±SD for the EMS Scale questions were 2.8±1.49, 3.31±1.58, 3.77±1.38, 3.28±1.44, 3±1.43, 2.83±1.4, and 2.81±1.6 for questions Q1 to Q7 respectively. The interval level was Neutral for all questions except Q3, which was Agree. The average marital satisfaction among mothers using the Enrich Marital satisfaction Scale is (3.11±0.58) which is considered a Neutral level. The mean of total score was (23.41±6.79) (Table 3).

**Table 3:** Mean, standard deviation, and the level of marital satisfaction among mothers using the EMS Scale

		Strongly disagree	**Disagree**	**Neutral**	**Agree**	**Strongly agree**	**Mean± SD**	**Direction**	**Level**
**Q**1	*N*	39	21	18	31	21	2.8±1.49	Neutral	Moderate
	%	30.0	16.2	13.8	23.8	16.2			
Q2	*N*	28	21	6	33	42	3.31±1.58	Neutral	Moderate
	%	21.5	16.2	4.6	25.4	32.3			
Q3	*N*	16	12	9	42	51	3.77±1.38	Agree	High
	%	12.3	9.2	6.9	32.3	39.2			
Q4	*N*	28	12	9	57	24	3.28±1.44	Neutral	Moderate
	%	21.5	9.2	6.9	43.8	18.5			
Q5	*N*	27	27	19	33	24	3±1.43	Neutral	Moderate
	%	20.8	20.8	14.6	25.4	18.5			
Q6	*N*	33	27	12	45	13	2.83±1.4	Neutral	Moderate
	%	25.4	20.8	9.2	34.6	10.0			
Q7	*N*	39	31	9	18	33	2.81±1.6	Neutral	Moderate
	%	30.0	23.8	6.9	13.8	25.4			
Weighted mean ± Std. Deviation							3.11±0.58	Neutral	Moderate
Total score							23.41±6.79		

### The A-REALD-30

The most recognized terms above 90% were (Pulp, Restoration, Denture, Gingiva, Analgesia, Sugar, Smoking, Extraction, and Caries). The mean (SD) values for the A-REALD were 21.65±6.02 (Table 4).

**Table 4:** Frequency, percentage, mean, and standard deviation of (A-REALD-30)

**A-REALD-30**	**Recognized (Score 1)**	**Unrecognized (Score 0)**	**Mean ±SD**
Temporomandibular	81 (62.3%)	49 (37.7%)	0.62±0.49
Hypoplasia	84 (64.6%)	46 (35.4%)	0.65±0.48
Plaque	87 (66.9%)	43 (33.1%)	0.67±0.47
Braces	96 (73.8%)	34 (26.2%)	0.74±0.44
Cellulitis	66 (50.8%)	64 (49.2%)	0.51±0.5
Apicoectomy	30 (23.1%)	100 (76.9%)	0.23±0.42
Fluoride	60 (46.2%)	70 (53.8%)	0.46±0.5
Bruxism	90 (69.2%)	40 (30.8%)	0.69±0.46
Pulp	118 (90.8%)	12 (9.2%)	0.91±0.29
Periodontal	57 (43.8%)	73 (56.2%)	0.44±0.5
Enamel	78 (60%)	52 (40%)	0.6±0.49
Restoration	127 (97.7%)	3 (2.3%)	0.98±0.15
Fistula	57 (43.8%)	73 (56.2%)	0.44±0.5
Sealant	60 (46.2%)	70 (53.8%)	0.46±0.5
Genetics	106 (81.5%)	24 (18.5%)	0.82±0.39
Incipient	81 (62.3%)	49 (37.7%)	0.62±0.49
Dentition	91 (70%)	39 (30%)	0.7±0.46
Abscess	112 (86.2%)	18 (13.8%)	0.86±0.35
Malocclusion	112 (86.2%)	18 (13.8%)	0.86±0.35
Denture	124 (95.4%)	6 (4.6%)	0.95±0.21
Gingiva	124 (95.4%)	6 (4.6%)	0.95±0.21
Hyperemia	78 (60%)	52 (40%)	0.6±0.49
Analgesia	121 (93.1%)	9 (6.9%)	0.93±0.25
Sugar	127 (97.7%)	3 (2.3%)	0.98±0.15
Smoking	124 (95.4%)	6 (4.6%)	0.95±0.21
Floss	90 (69.2%)	40 (30.8%)	0.69±0.46
Extraction	127 (97.7%)	3 (2.3%)	0.98±0.15
Halitosis	70 (53.8%)	60 (46.2%)	0.54±0.5
Caries	121 (93.1%)	9 (6.9%)	0.93±0.25
Temporomandibular	81 (62.3%)	49 (37.7%)	0.62±0.49
Total Score of REALD	Mean ±SD	Range	
	21.65±6.02	3 - 29	

### Correlations between OHL, MS and clinical indices

Sperman’s correlation test between the total marital satisfaction score and the other tested parameters (A-REALD-30, DMFT, dmft, PI, and GI revealed a weak negative relation with all except a weak positive relation with A-REALD-30, *p* value= 443, and a moderate negative relation with DMFT of the mothers *p* value= 004 (Table 5). As for the correlation between A-REALD-30 total score and different parameters (DMFT, dmft, PI, and GI), a weak positive relation was reported, *p* value= o.549, 0.412, with the DMFT and dmft respectively. Whereas a weak negative relation was shown with the GI and PI of the mothers, *p* value= 0.670 and 0.158, respectively (Table 6).

**Table 5:** Correlation between the total marital satisfaction score with A-REALD-30, DMF, dmf, PI, and GI

	r^**^	***P-value***	**Correlation type**
Marital satisfaction vs. A-REALD-30	0.068	0.443^NS^	Weak positive
Marital satisfaction vs. DMF	−0.253	0.004^S^	Moderate Negative
Marital satisfaction vs. dmf	−0.188	0.032^S^	Weak Negative
Marital satisfaction vs. PI	−0.005	0.956^NS^	Weak Negative
Marital satisfaction vs. GI	−0.031	0.730^NS^	Weak Negative

- ^**^Spearman Correlation value

-*S* Significant (Correlation is significant at the 0.05 level)

-*NS* Non-significant (Correlation is insignificant at the 0.05 level)

-*HS* Highly significant (Correlation is significant at the 0.001 level)

**Table 6:** Correlation between A-REALD-30 total score with DMF, dmf, PI, and GI

	r^**^	***P*-value**	**Correlation type**
A-REALD-30 vs. DMF	0.053	0.549^NS^	Weak Positive
A-REALD-30 vs. dmf	0.073	0.412^NS^	Weak Positive
A-REALD-30 vs. PI	−0.125	0.158^NS^	Weak Negative
A-REALD-30 vs. GI	−0.038	0.670^NS^	Weak Negative

-^**^Spearman Correlation value

-*S* Significant (Correlation is significant at the 0.05 level)

-*NS* Non-significant (Correlation is insignificant at the 0.05 level)

-*HS* Highly significant (Correlation is significant at the 0.001 level)

## Discussion

To improve personal and community health outcomes and to permit the formulation of health promotion strategies, it is essential to focus on different cultural and social determinants of the OH [3, 29]. This cross-sectional study is the first to evaluate the effect of mother’s marital satisfaction and OHL on their own and their preschool children’s oral health status. In the Egyptian society, mothers usually are primarily responsible for issues regarding their children

particularly those related to health and well-being, this role is influenced by the Egyptian culture and caregiving norms, this is why we chose to focus on mothers in our study because they’re the primary influence in matters of the children’s health.

In this work, the mean EMS total score of mothers evaluated in Cairo regional unit was 23.41. Only one study reported a total EMS score of (45.85) for mothers in the Upper Egypt regional unit [30]. The previous 2 scores are not comparable due to different study areas with different social and demographic context, different participant criteria, and even different versions of the EMS scale. In the Middle East, the EMS for a sample of Iranian women was 37.17 in a version that used 10 questions of the original EMS scale [25]. Several socioeconomic and demographic factors are known to affect levels of marital well-being such as religious beliefs, sexual practices, communicative and interactive skills, mental health, couple’s occupation, marriage duration, number of children, and family income [25].

The total score of REALD-30 in this work was 21.65 ranging from 3–29, slightly lower than that reported by Badran et al., 2023 (25.9 ranging from 3–30) [3]. This reflects generally a low level of OHL in Egypt.

The evaluation of the impact of both OHL and MS on oral health characteristics of individuals cannot be done independently of other factors such as levels of education, source of oral health information, area of residence, and oral health-related behaviours of the participants. OHL was shown to be significantly associated with the level of education of patients [31, 32], others reported otherwise [33]. The same applies to the relationship between marital satisfaction and mothers’ education [34], as a non-consistent conclusion on this relation was reported, some argue that education level has a significant effect on marital satisfaction [35], while others reported no relation [36]. To minimize the effect of variable education levels and ensure that they can adequately read and comprehend the questions, we chose well-educated mothers with secondary and university degrees to be included in this work. Although the highly educated sample may potentially limit the range of responses to OHL skills, the total score of REALD −30 was 21.65 ranging from 3–29; indicating that Egyptian mothers with a good level of education, have low levels of OHL. In accordance, the sum of the REALD-30 score in Chinese-educated parents was 23.91 [37]. Further, in Indian highly educated mothers, 57% had Low (≤21) OHL scores and 30% reported scores less than 26 [31].

Health literacy is a group of cognitive and social skills that demonstrate a person’s motivation and ability to find, understand, and appropriately use health information. While many sources of health information are widely accessible, people have different social contexts and preferences for health information sources. Our descriptive data showed that the Internet was the most frequently utilized source of oral health information among mothers (41.5%), 18.5% reported media (television and radio), and only 11.5% reported healthcare-related personnel. Information from health-related personnel is a vital source of health information as it is significantly associated with higher general health literacy [38].

Dental plaque accumulation is positively correlated with caries and periodontal diseases, both diseases are prevented by frequent teeth brushing at least twice daily [39]. Further, Parents’ oral health behavior has been established to affect their children’s oral health behavior and oral health status [37, 40]. The mean PI for mothers was 1.29 and 30% of them reported brushing their teeth twice a day, however, only 16% of the children brush with the same frequency, indicating that children don’t brush their teeth as frequently as their mothers. Similar findings were reported by Yu Wang etal., 2022 [37].

Further, 93% of mothers visit the dentist only when feeling pain and 4.6% never visited a dentist, the reported low levels of OHL can justify this finding. Previous studies have suggested that low OHL leads to a decreased adherence to positive oral health behaviors [41], and regular visitors to a dentist had higher levels of OHL than those visiting only when they felt pain [33]. However, others did not find an association between OHL and the utilization of dental services [42]. Whether the reported low OHL of mothers comes as a result of infrequent visits to the dentist, or the infrequent visits deprive the mothers of obtaining the knowledge that promotes the OHL, this remains to be assessed.

Dental caries in children is largely dependent upon their dietary habits, oral hygiene, and fluoride exposure [43], the frequency of sugar intake and daily brushing were included in the questionnaire, whereas, the REALD-30 included fluoride and sealant words; the two words were among the highly unrecognized terms among mothers. Further, nearly 65% of children consume sugar daily and 26% consume sugar more than 3 times daily. Egyptian adolescents and preschool children hold a relatively high dmft score, especially in civilized areas like Cairo [44]. In this work, the mean of decayed, missed, and filled teeth indices for mothers and children were 5.07 and 5.42 respectively, slightly higher than those reported earlier in 2022 (4.68 and 4.82 respectively) [44, 45]. This is considerably higher compared to the Indian preschool children’s mean score (2.61) [46] However, the different cultural and social influences of each population cannot be ignored.

In this work, for mothers and children, a weak positive correlation between the REALD-30 scores and both DMFT and dmft was shown, in contrast with others, who reported a negative association between the two variables [46, 47]. However, conflicts in this regard exist, a study on preschool children in Brazil found no association between OHL of parents and children’s caries index and suggested that OHL in one way or another overlaps socioeconomic status (income, parent’s education, number of siblings etc) [48]. The discrepancy in result could be further supported by the fact that our study participants are low-income families who are seeking free dental treatment in educational facilities; thus, the effects of other socioeconomic variables such as family income cannot be denied. In accordance with this explanation, a previous study showed that dental caries sores in Egyptian preschool children had significant inverse relations with family income [45]. Further, our study reported a negative association between a social determinant like marital satisfaction and both the DMFT and dmft, and the PI of mothers indicating that the OHL of parents is not the sole player in the context of children’s oral health.

It has been established that individuals with good literacy have better oral hygiene status [49, 50]. In this work, a weak negative relation between the mother’s PI, GI, and REALAD-30 was shown, in agreement with previous data [51, 52]. Finally, our reported discrepancy in the relations between REALD and both the DMFT and dmft (positive) and the gingival and plaque indices (negative), highlights the limitations of using REALD-30 alone as a data collection tool for measuring OHL as it only tests word recognition and pronunciation, not the meaning of the words [48]. Using a comprehension instrument showed a stronger association with children’s oral status than a word recognition instrument when assessing caregivers’ OHL [53]**.** Further, data reported that OHL was not associated with dental health knowledge or behaviors, and assumed that oral health behavior is as valuable as knowledge -if not more- in predicting oral health outcomes [3, 22].

The effect of socioeconomic inequalities on oral health has been highly acknowledged, for example, dental caries was significantly associated with socioeconomic variables like family income and the number of siblings [54]. Our study showed that Mother’s marital satisfaction was negatively associated (weak to moderate association) with all studied indices (DMFT, dmft, gingival, and plaque indices). Our results can be interpreted by the known effects of marriage on health behaviors, mental health, intergenerational health effects, and health care access, use, and cost [55]. Indeed, these effects on general health can also apply to oral health. Having a satisfying marriage results in fewer physical and mental health problems for mothers and their children due to better handling of life’s pressures, this can affect the oral health related behaviors and attitudes like teeth brushing, eating healthy diet, frequent visits to dental clinics, better utilization of oral health services and most important to the context of this work; the possible intergenerational health as parental marital status may have long-term oral health consequences for children. Indeed, all the previously mentioned possible effects lack adequate evidence and need to be investigated. Finally, individuals with mental affection are prone to have poor oral health, although the nature of this relationship is not fully explained health [7, 9].

Finally, a weak positive non-significant relation was shown between REALD-30 and EMS, in accordance with previous data found a significant positive relationship between health literacy and marital satisfaction of married women [56]. This could be a true association, or it could be biased by socially desirable responses in the context of the questionnaire we used in the clinical settings. A more thorough evaluation of oral health behaviors may be needed to understand the association between mothers’ literacy and or marital satisfaction and child health outcomes utilizing common factors to assess the causal relation between the tested variables.

## Conclusions

Within the study’s limitations, our study suggests that marriage could relate to the oral health outcomes, however, the picture is not yet complete. Future research could further explore the effects of marriage on oral health care costs; the oral health effects of marriage for different socio-economic groups, and the intergenerational health effects of marriage. Better information about how marriage affects oral health can add to discussions about the role and purposes of public policy in supporting marriage. Moreover, it can be concluded that OHL is not the only factor in determining the oral health status of mothers and children. Other socioeconomic determinants like education, family income, source of oral health information, OH attitude, and practice should be considered when planning community dental services. Finally, REALD-30 should be used with more sophisticated assessment tools for more in-depth analysis.

## Limitations

Our findings should be considered within the context of certain limitations. First, the cross-sectional design does not allow for establishing temporal relations and causal reasoning between factors and outcomes**.** A reverse causation was possible as some mothers may have recognized certain dental words during prior treatment for their children. Second, the limitations of the instruments used. Third, the sample was not completely representative of the Egyptian population due to study location in the great Cairo regional unit and the inclusion of secondary or higher education may limit the study’s external validity for less-educated populations. Fourth, As with any study relying on self-reported data, there is a risk of subjectivity and response bias, future studies with tools that reduce this bias are needed. A longitudinal design would be needed to determine if parental health literacy and state of marital well-being relate to their child's future dental health.

## Supplementary Information

Supplementary Material 1.

Supplementary Material 2.

Supplementary Material 3.

## Data Availability

The datasets generated and/or analyzed during the current study are available from the corresponding author on reasonable request

## Abbreviations

OHL: Oral health literacy
MS: Marital Satisfaction
EMS: ENRICH Marital Satisfaction
A-REALED-30: Arabic Rapid Estimate of Adult Literacy in Dentistry
DMFT: Decayed, Missed, Filled Teeth
PI: Plaque index
GI: Gingival index
