# Supplementary material for: Oral health status of Egyptian mothers and their preschool children: association of mother’s oral health literacy and marital satisfaction- a cross-sectional study
Source: BMC Oral Health. 2025 May 22;25:767. doi: 10.1186/s12903-025-06099-8 (PMC12096523; doi:10.1186/s12903-025-06099-8)

| **Descriptive Statistics** | | | |
| --- | --- | --- | --- |
|  | Mean | Std. Deviation | N |
| AREALD Score | 21.6462 | 6.02303 | 130 |
| Marital_Score | 23.4077 | 6.78770 | 130 |

| **Correlations** | | | |
| --- | --- | --- | --- |
|  | | AREALD Score | Marital_Score |
| Spearman Correlation | AREALD Score | 1.000 | .070 |
|  | Marital_Score | .070 | 1.000 |
| Sig. (1-tailed) | AREALD Score | . | .443 |
|  | Marital_Score | .443 | . |
| N | AREALD Score | 130 | 130 |
|  | Marital_Score | 130 | 130 |

| **Variables Entered/Removed^a^** | | | |
| --- | --- | --- | --- |
| Model | Variables Entered | Variables Removed | Method |
| 1 | Marital_Score^b^ | . | Enter |

| a. Dependent Variable: AREALD Score |
| --- |
| b. All requested variables entered. |

| **Model Summary^b^** | | | | |
| --- | --- | --- | --- | --- |
| Model | R | R Square | Adjusted R Square | Std. Error of the Estimate |
| 1 | .070^a^ | .005 | -.003 | 6.03180 |

| a. Predictors: (Constant), Marital_Score | | | | |  |  |  |
| --- | --- | --- | --- | --- | --- | --- | --- |
| b. Dependent Variable: AREALD Score | | | | |  |  |  |
| **ANOVA^a^** | | | | | | | |
| Model | | Sum of Squares | df | Mean Square | | F | Sig. |
| 1 | Regression | 22.755 | 1 | 22.755 | | .625 | .430^b^ |
|  | Residual | 4656.968 | 128 | 36.383 | |  |  |
|  | Total | 4679.723 | 129 |  | |  |  |

| a. Dependent Variable: AREALD Score |
| --- |
| b. Predictors: (Constant), Marital_Score |

| **Coefficients^a^** | | | | | | |
| --- | --- | --- | --- | --- | --- | --- |
| Model | | Unstandardized Coefficients | | Standardized Coefficients | t | Sig. |
|  |  | B | Std. Error | Beta |  |  |
| 1 | (Constant) | 20.198 | 1.906 |  | 10.595 | .000 |
|  | Marital_Score | .062 | .078 | .070 | .791 | .430 |

| a. Dependent Variable: AREALD Score |
| --- |

| **Residuals Statistics^a^** | | | | | |
| --- | --- | --- | --- | --- | --- |
|  | Minimum | Maximum | Mean | Std. Deviation | N |
| Predicted Value | 20.6309 | 22.3634 | 21.6462 | .42000 | 130 |
| Residual | -19.36344 | 7.75033 | .00000 | 6.00837 | 130 |
| Std. Predicted Value | -2.417 | 1.708 | .000 | 1.000 | 130 |
| Std. Residual | -3.210 | 1.285 | .000 | .996 | 130 |

| a. Dependent Variable: AREALD Score |
| --- |

**Charts**


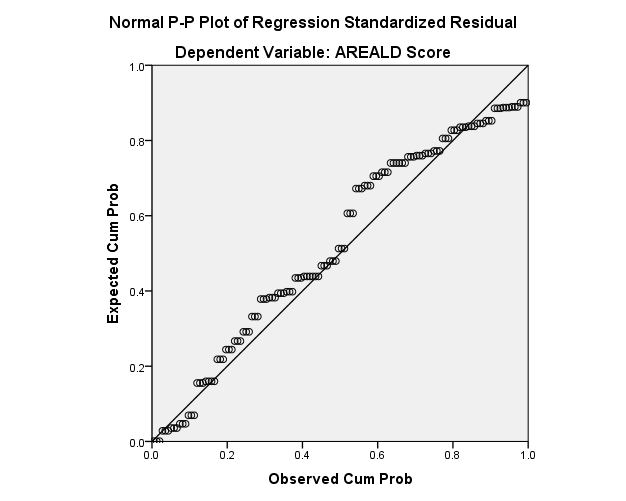


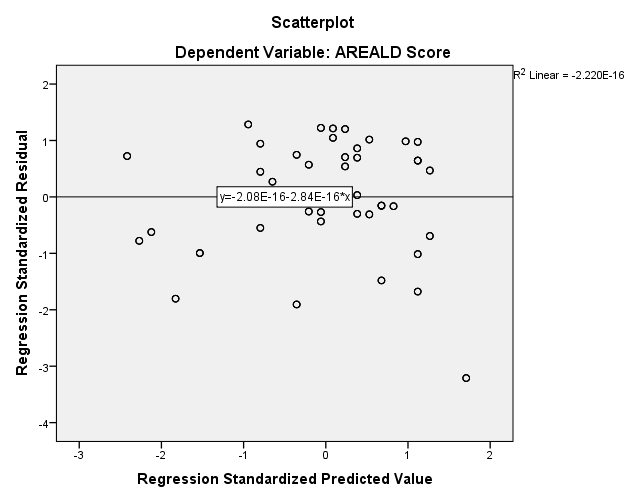


We opted not to apply the **linear regression test** to our results because the **Spearman correlation coefficient test** revealed a very weak correlation between the different parameters, particularly between **RELAD** and **Marital Satisfaction**. The Spearman correlation test is capable of detecting **monotonic non-linear relationships**, whereas the linear regression test is limited to **linear relationships** and may yield misleading conclusions if applied to non-linear data (1). Therefore, to avoid drawing false conclusions, we preferred the Spearman correlation test, as it provides detailed information about the **direction of the correlation** (positive or negative), the **strength of the correlation** (weak, moderate, or strong), and the **significance** (significant or non-significant).

Nevertheless, we proceeded to apply the **linear regression test** to the results of **RELAD** and **Marital Satisfaction**. The regression analysis indicated a linear relationship with a very weak **R² value (0.005)**, which is entirely consistent with the findings of the Spearman correlation test. The earlier Spearman results had already indicated a very weak correlation that was not statistically significant. Thus, we can conclude that the results of the linear regression align with the Spearman correlation findings, further supporting the absence of a meaningful relationship between RELAD and Marital Satisfaction.


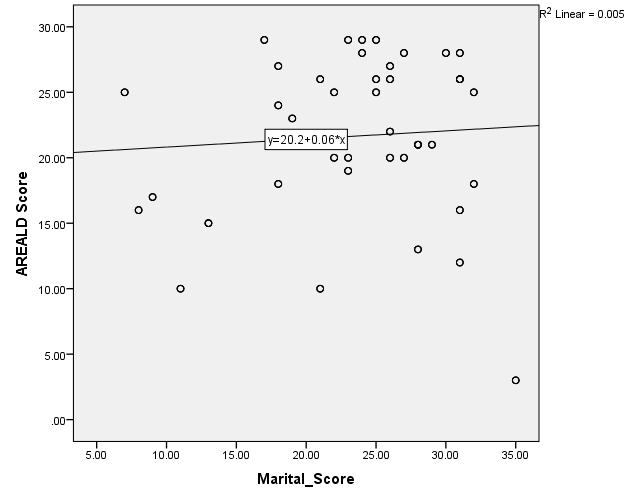

Supplement: Supplementary file 1 — Supplementary Material 1. [file 12903_2025_6099_MOESM1_ESM.docx]
