# Supplementary material for: Oral health status of Egyptian mothers and their preschool children: association of mother’s oral health literacy and marital satisfaction- a cross-sectional study
Source: BMC Oral Health. 2025 May 22;25:767. doi: 10.1186/s12903-025-06099-8 (PMC12096523; doi:10.1186/s12903-025-06099-8)
Supplement: Supplementary file 2 — Supplementary Material 2. [file 12903_2025_6099_MOESM2_ESM.docx]

**Oral Health Status of Egyptian Mothers and Their Preschool Children: Association of Mother’s Oral Health Literacy and Marital Satisfaction - A Cross-Sectional Study**

**Section 1: Personal data**:

- **Mother’s age: •** ≤ 30 **•** > 30
- **Child’s age:** …………….
- **Child’s sex: •** Male **•** Female
- **Area of residence: •** Urban **•** Rural
- **Mother’s occupation: •** Working **•** Nonworking
- **Mother’s education level: •** Secondary school **•** University degree
- **The source of oral health information:**

**•** Me **•** Books **•** Internet **•** Health service **•** Others **•** Nothing

- **Brushing of the child’s teeth: •** Up to 1 **•** ≥2 **•** Irregular **•** No brushing
- **Brushing of the mother’s teeth: •** Up to 1 **•** ≥2 **•** Irregular **•** No brushing
- **Child’s sugar consumption: •** Irregular **•** ≤3 times/day **•** >3 times/day
- **How often does the mother visit the dentist?**

**•** Regular **•** Only with complaints **•** Didn’t see the dentist

**Section 2: The level of marital satisfaction among mothers using the Enrich Marital Satisfaction Scale:**

1. **I am not pleased with the personality and characteristics and personal habits of partner.**

**•** Strongly agree **•** Moderately agree **•** Neither agree nor disagree **•** Moderately disagree **•** Disagree

1. **I am very happy with how we handle role responsibilities in our marriage.**

**•** Strongly agree **•** Moderately agree **•** Neither agree nor disagree **•** Moderately disagree **•** Disagree

1. **Our relationship is a perfect success.**

**•** Strongly agree **•** Moderately agree **•** Neither agree nor disagree **•** Moderately disagree **•** Disagree

1. **I am very happy about how we make decisions and resolve conflicts.**

**•** Strongly agree **•** Moderately agree **•** Neither agree nor disagree **•** Moderately disagree **•** Disagree

1. **I am unhappy about our financial position and the way we make financial decision.**

**•** Strongly agree **•** Moderately agree **•** Neither agree nor disagree **•** Moderately disagree **•** Disagree

1. **I have some needs that are not being met by our relationship.**

**•** Strongly agree **•** Moderately agree **•** Neither agree nor disagree **•** Moderately disagree **•** Disagree

1. **I am not satisfied with the way we each handle our responsibilities as parents.**

**•** Strongly agree **•** Moderately agree **•** Neither agree nor disagree **•** Moderately disagree **•** Disagree

**Section 3: Rapid Estimate of Adult Literacy in Dentistry (A-REALD-30):**

| **A-REALD-30** |  |
| --- | --- |
| **Temporomandibular** |  |
| **Hypoplasia** |  |
| **Plaque** |  |
| **Braces** |  |
| **Cellulitis** |  |
| **Apicoectomy** |  |
| **Fluoride** |  |
| **Bruxism** |  |
| **Pulp** |  |
| **Periodontal** |  |
| **Enamel** |  |
| **Restoration** |  |
| **Fistula** |  |
| **Sealant** |  |
| **Genetics** |  |
| **Incipient** |  |
| **Dentition** |  |
| **Abscess** |  |
| **Malocclusion** |  |
| **Denture** |  |
| **Gingiva** |  |
| **Hyperemia** |  |
| **Analgesia** |  |
| **Sugar** |  |
| **Smoking** |  |
| **Floss** |  |
| **Extraction** |  |
| **Halitosis** |  |
| **Caries** |  |
| **Temporomandibular** |  |
| **Total Score of REALD: ……… Out of 30** | |
